# New training model using chickens intestine for pediatric intestinal anastomosis^1^

**DOI:** 10.1590/s0102-865020190070000009

**Published:** 2019-09-16

**Authors:** Deivid Ramos dos Santos, Faustino Chaves Calvo, Daniel Haber Feijó, Nayara Pontes de Araújo, Renan Kleber Costa Teixeira, Edson Yuzur Yasojima

**Affiliations:** I Fellow Master degree, Postgraduate Program in Surgery and Experimental Research, Universidade do Estado do Pará (UEPA), Belem-PA, Brazil. Acquisition and interpretation of data; conception, design, intellectual and scientific content of the study; interpretation of data; manuscript writing; II Graduate student, School of Medicine, UEPA, Belem-PA, Brazil. Acquisition and interpretation of data, manuscript writing; III Fellow Master degree, Postgraduate Program in Surgery and Experimental Research, UEPA, Belem-PA, Brazil. Acquisition and interpretation of data; IV MS, Department of Experimental Surgery, School of Medicine, UEPA, Belem-PA, Brazil. Interpretation of data, statistics analysis, critical revision; V PhD, Associate Professor, Department of Experimental Surgery, School of Medicine, UEPA, Belem-PA, Brazil. Conception, design, intellectual and scientific content of the study, final revision

**Keywords:** Anastomosis, Surgical, Training, Surgical Procedures, Operative

## Abstract

**Purpose:**

To develop a new low-cost, easy-to-make and available training model using chickens’ intestine for infant intestinal anastomosis.

**Methods:**

Segments of chicken intestine were used to create an intestinal anastomosis simulator. We tried to perform an end-to-end, end-to-side and side-to-side anastomosis. Handsewn sutured anastomosis were performed in single layered with interrupted prolene 5-0 suture. The parameters analyzed were cost, intestine's diameter and length, anastomosis patency and flow-through and leakage amount.

**Results:**

In all cases it was possible to make the anastomosis in double layered without difficulties, different from the usual ones. There was a positive patency at all anastomoses after the end of the procedure, with no need for reinterventions.

**Conclusion:**

The new training model using chickens’ intestine for infant intestinal anastomosis is low-cost, easy-to-make and easy available.

## Introduction

Intestinal anastomosis becomes necessary when the segment on the gastrointestinal tract is resected for benign or malignant conditions and gastrointestinal continuity needs to be restored^1^. Failure of an anastomosis with leakage of intestinal contents is one of the most significant surgical complications^1^ ^,^ ^2^. Reported failure rates range from 1 to 24%, depending of the surgeon's experience, what type of anastomosis was performed and whether the operation was an elective or an emergency procedure^1^ ^,^ ^3^ ^,^ ^4^.

The surgeon experience is based on what technique will be used according to disease process and on the caliber of intestinal structure; intestinal wall diameters in small bowel and colon range from 0.7 to 1.1mm and 1.0 to 1.4mm, respectively^5^. Especially in children who have smaller structures, specific training, non-biological or biological models (live or non-living) instead surgical training directly in human, is necessary to improve pediatric intestinal anastomoses and minimize complications^6^ ^,^ ^7^.

The use of simulators facilitates the development of initial surgical skills that will be transposed into clinical practice, reducing surgical time, number of complications, length of stay and morbidity and mortality^6^ ^–^ ^9^. In recent years, there has been a shift towards high-fidelity simulation involving advanced technology such as virtual reality and 3-D printed models^10^. Nevertheless, the high cost limits the access to high-income tertiary centers^6^ ^,^ ^7^ ^,^ ^10^ ^,^ ^11^.

In relation to pediatric surgery, there is a difficulty in the creation of simulators due to factors such as different stages of growth of the child and small size of the structures^8^ ^,^ ^9^. So, the aim of this paper is to develop a new low-cost, easy-to-make and easily available low-fidelity training model using chickens’ intestine for infant intestinal anastomosis.

## Methods

The research followed the rules of the Brazilian Law for Animal Care (Law: 11.794/08) that is based on NIH guidelines, and followed the rules of Council for International Organization of Medical Sciences ethical code for animal experimentation and the European Convention for the Protection of Vertebrate Animals Used for Experimental and Other Scientific Purposes.

Chicken intestines were obtained at a butcher's shop following the Brazilian and local sanitary regulations. There was no acquisition cost. Normally, the chicken intestines are discarded, because they are not used in the feeding of the local population.

**Figure 1:**
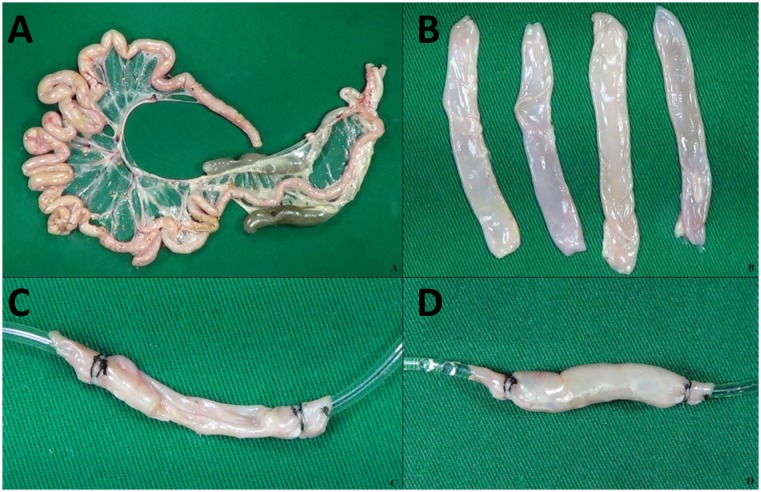
Main maker steps. **A** - Chicken's intestine. **B** - Bowel fragments after wash. **C** - Model connected with infusion set. **D** - Initial patency test.

The steps of the training system creation are shown in Figure 1. First, we removed the mesentery and colons. The intestine was inverted (inside-out) and washed with running water and soap to remove better intestinal contents, minimizing deterioration and smell. Afterwards, it was inverted again (normal position) and was sectioned into 8 cm segments. It was inserted into each end an infusion set and fixed with 2-0 silk. One side is block, and was tested the initial patency and presence of holes. So, the training system was finished and was ready to be used to simulate superficial or deep (with a box, such a shoe box) anastomosis.

We tried to perform an end-to-end, end-to-side and side-to-side anastomosis. All anastomoses were performed by two experienced general surgeons with more than 5 years of experience. Handsewn sutured anastomosis were performed in single layered^10^ ^,^ ^12^ with an interrupted prolene 5-0 suture.

**Figure 2:**
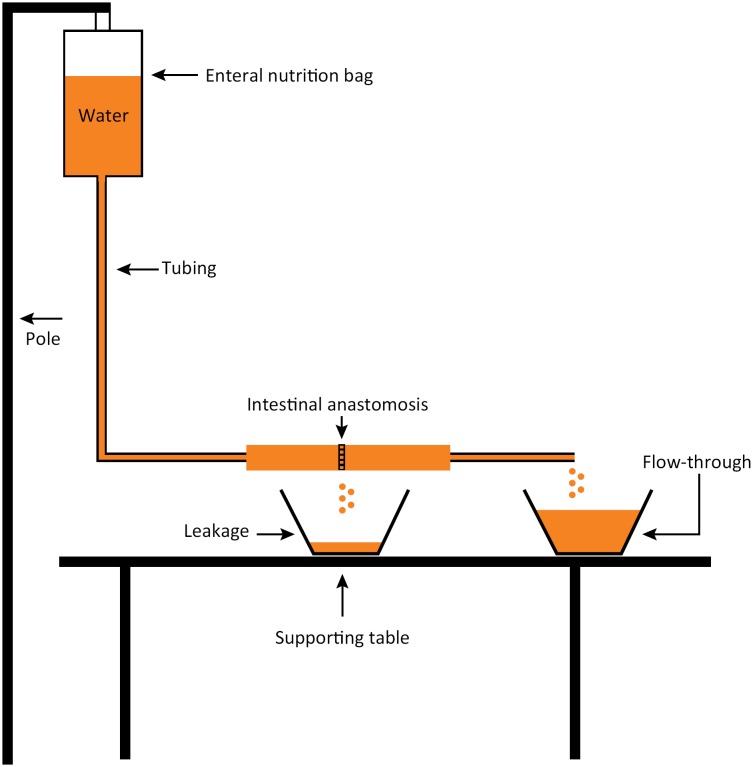
Diagram of testing apparatus.

To objectively measure the amount of stenosis and leakage, we used a system that allowed water to flow through the anastomosis^13^ (Fig. 2). The system consists of an enteral nutrition bag filled with 20 mL of water, hanging on a pole 20 cm above the tabletop, connected with an infusion set cut at the tip. The end of infusion set was inserted into one side of the simulated anastomosis and fixed with 2-0 silk. The other end was connected and fixed to an extension tubing with an open end. The intestine was suspended above a plastic container and the open end was allowed run into a second plastic container. The stopcock was opened, and water could run through the intestine for 60 seconds. The amount of water collected in the container situated under the anastomosis represented leakage and the amount collected in the second container determined by the amount of water flow-through. Prior to the start of each training trial, both containers were emptied, dried with a towel, and weighed to ensure accurate starting measurements.

The parameters analyzed were: 1) Cost (in dollar); 2) Intestine's diameter and length (cm); 3) Completion time (min); 4) Anastomosis patency (positive or negative); and 5) Flow-through amount and Leakage amount (in mL). The results were presented as mean ± standard error of the mean.

## Results

The diameter and length of the chicken intestine used ranged from 0.5 to 3 cm (mean: 2.08 ±0.77 cm) and 100 to 120 cm (mean: 108.44 ±7.59 cm), respectively. On average, 12 simulators were produced with one bowel. The average time for making a simulator was 9.22 ±1.45 minutes. All models showed no signs of deterioration within 30 days of observation when stored under refrigeration. The cost of each simulator was approximately U$ 3.80.

**Figure 3:**
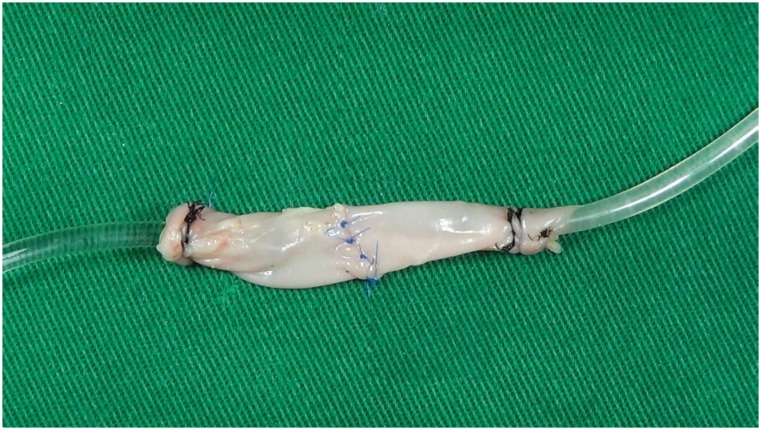
Finished end-to-end anastomosis.

In all cases it was possible to make the anastomosis without difficulties unlike the usual ones (Fig. 3). There was a positive patency at all anastomosis after the end of the procedure, with no need for reinterventions. The average time to perform an end-to-end anastomosis was 25.79 ±4.44 minutes, it was 30.77 ±3.86 minutes on end-to-side anastomosis, and side-to-side anastomosis mean time was 29.92 ±4.12.

The mean flow-through was 19.71 ±0.21ml for end-to-end anastomosis, 19.55 ±0.26ml for end-to-side anastomosis and 19.28 ±0.33ml for side-to-side anastomosis. The mean leakage amount was 0.16 ±0.04ml for end-to-end anastomosis, 0.25 ±0.06ml for end-to-side anastomosis and 0.24 ±0.05ml for side-to-side anastomosis.

## Discussion

Pediatric surgeons must have a versatile technical skill set, to address several surgical sites (gastrointestinal, thoracic, urologic surgery, and others)^7^ ^,^ ^10^. To develop these abilities, it is necessary a sustained practice and a continuous education input. The old model of surgical training based on the maximal “see one, do one, teach one” has been increasingly questioned^10^ ^,^ ^14^. Progressively, there is a change to a constant, and initially supervised, training model. This change stems from the high rates of morbidity and mortality and an increase in the length of hospital stay that occurred due to the initial practice directly in humans^7^ ^,^ ^8^ ^,^ ^11^.

These facts have led to an increase in the need for simulation-based education. The firsts steps were performed with simple and low-realistic models for training the surgical techniques, instruments’ handling and manual dexterity. In some degree, the use of non-live high-realistic models is highlighted, because it possibly reproduces all main stages of the surgery or procedure, even as simulate complications and anatomical variations. In the end of the learning curve, the use of live models is common, in which it is possible to perform all steps of the surgery and the follow-up can be evaluated. All theses phases are necessary to ensure the safety of surgeries or procedures, especially when performed on children and infants^7^ ^–^ ^11^ ^,^ ^13^ ^,^ ^14^.

In this study, we developed and validated an inanimate high-realistic training model to simulate infant intestinal anastomoses, as as it allows to teach medical students and surgical residents the practice of all kinds of anastomoses (end-to-end, end-to-side and side-to-side). This model may be preferred to live models because the chicken intestine is readily available, low cost, does not require any special treatment, is highly similar to intestine segments and respects the ethical principle of reducing the unnecessary use of animals^6^ ^–^ ^10^.

Some advantages of this training model are: possibility of determining the size to be used (larger caliber - 3 to 5 cm - for beginners; and smaller caliber - 0.5cm - for experienced students); the training could be evaluated by objective parameters, such as: anastomosis time (long time indicates low domain of the technique), number of knots and throws per knot (low number indicates low domain of the technique), flow-through^3^ (higher flow-through indicates little to no vessel stenosis) and leakage amount^3^ (lower leakage indicates little to no vessel leakage at the anastomosis location); and it is portable and homemade, allowing its use outside of laboratories.

In this simulator, the using staplers to perform the anastomosis is a limitation^5^, as what happens in infants, because the staples are too big, implicating them as a causative factor in anastomosis leakage. Reinforcing the importance of hand-sewn anastomosis training^1^ ^,^ ^2^ ^,^ ^10^, as well as being able to be used in the future for the training of stapled anastomoses, with staplers better adapted to the caliber of infant’ intestine. In this study, we performed a double layered anastomosis; however, it is possible to simulate different kinds of anastomoses (double layered, end-to-side or side-to-side)^2^ ^,^ ^6^ ^,^ ^10^ in this training model.

Three-dimensional printing is an efficient and affordable way to create simulation models. 3D models for procedure learning and competency assessment are available to model anatomy, practice procedures and plan surgical interventions^15^. However, these simulations are largely inaccessible to most healthcare systems, due to the high-cost. To enhance accessibility of surgical simulation in source-poor settings, we have developed this low-cost, easily reproducible model. Beyond basic surgical instruments, our model requires animal tissues which would be accessible within the locality of the healthcare system.

The findings of our study should be interpreted within the context of its limitations. This model could not evaluate the late post-surgery (anastomosis leakage, necrosis, paralytic ileus and others); however, the usage of flow-through and leakage amount could be used to indicate technical failure the main cause of initial complications.

## Conclusions

The new training model using chickens’ intestine for infant intestinal anastomosis is low-cost, easy-to-make and easily available. This model could be used for practice end-to-end, end-to-side and side-to-side anastomosis, improving intestinal anastomosis skills on most metrics by engaging in simulation-based training.
